# Association of Plasma Neurofilament Light With Neurodegeneration in Patients With Alzheimer Disease

**DOI:** 10.1001/jamaneurol.2016.6117

**Published:** 2017-03-27

**Authors:** Niklas Mattsson, Ulf Andreasson, Henrik Zetterberg, Kaj Blennow

**Affiliations:** 1 Clinical Memory Research Unit, Department of Clinical Sciences, Faculty of Medicine, Lund University, Lund, Sweden; 2 Memory Clinic, Skåne University Hospital, Scania, Sweden; 3 Department of Neurology, Skåne University Hospital, Scania, Sweden; 4 Clinical Neurochemistry Laboratory, Sahlgrenska University Hospital, Mölndal, Sweden; 5 Department of Psychiatry and Neurochemistry, Institute of Neuroscience and Physiology, Sahlgrenska Academy at the University of Gothenburg, Möndal, Sweden; 6 Department of Molecular Neuroscience, University College London Institute of Neurology, Queen Square, London, England

## Abstract

**Importance:**

Existing cerebrospinal fluid (CSF) or imaging (tau positron emission tomography) biomarkers for Alzheimer disease (AD) are invasive or expensive. Biomarkers based on standard blood test results would be useful in research, drug development, and clinical practice. Plasma neurofilament light (NFL) has recently been proposed as a blood-based biomarker for neurodegeneration in dementias.

**Objective:**

To test whether plasma NFL concentrations are increased in AD and associated with cognitive decline, other AD biomarkers, and imaging evidence of neurodegeneration.

**Design, Setting, and Participants:**

In this prospective case-control study, an ultrasensitive assay was used to measure plasma NFL concentration in 193 cognitively healthy controls, 197 patients with mild cognitive impairment (MCI), and 180 patients with AD dementia from the Alzheimer’s Disease Neuroimaging Initiative. The study dates were September 7, 2005, to February 13, 2012. The plasma NFL analysis was performed in September 2016.

**Main Outcomes and Measures:**

Associations were tested between plasma NFL and diagnosis, Aβ pathologic features, CSF biomarkers of neuronal injury, cognition, brain structure, and metabolism.

**Results:**

Among 193 cognitively healthy controls, 197 patients with mild cognitive impairment, and 180 patients with AD with dementia, plasma NFL correlated with CSF NFL (Spearman ρ = 0.59, *P* < .001). Plasma NFL was increased in patients with MCI (mean, 42.8 ng/L) and patients with AD dementia (mean, 51.0 ng/L) compared with controls (mean, 34.7 ng/L) (*P* < .001) and had high diagnostic accuracy for patients with AD with dementia vs controls (area under the receiver operating characteristic curve, 0.87, which is comparable to established CSF biomarkers). Plasma NFL was particularly high in patients with MCI and patients with AD dementia with Aβ pathologic features. High plasma NFL correlated with poor cognition and AD-related atrophy (at baseline and longitudinally) and with brain hypometabolism (longitudinally).

**Conclusions and Relevance:**

Plasma NFL is associated with AD diagnosis and with cognitive, biochemical, and imaging hallmarks of the disease. This finding implies a potential usefulness for plasma NFL as a noninvasive biomarker in AD.

## Key Points

**Question:**

What is the importance of plasma neurofilament light in Alzheimer disease?

**Findings:**

In this case-control study of 193 cognitively healthy controls, 197 patients with mild cognitive impairment, and 180 patients with Alzheimer disease dementia, plasma neurofilament light was associated with Alzheimer disease and correlated with future progression of cognitive decline, brain atrophy, and brain hypometabolism.

**Meaning:**

Plasma neurofilament light may be a promising noninvasive biomarker for Alzheimer disease.

## Teaser

This case-control study compares patients with mild cognitive impairment, patients with AD dementia, and cognitively healthy patients to test whether plasma neurofilament light concentrations are increased in patients with AD and associated with cognitive decline, other AD biomarkers, and imaging evidence of neurodegeneration.

## Introduction

Alzheimer disease (AD) is a neurodegenerative disease that is characterized by brain accumulation of β-amyloid (Aβ) and tau, progressive atrophy, and cognitive decline. Biomarkers that capture biological processes in AD are increasingly used to support the diagnosis of AD in research, drug development, and clinical practice.^1^ The most well-established AD biomarkers include structural magnetic resonance imaging (MRI); cerebrospinal fluid (CSF) biomarkers of Aβ, tau, and neuronal injury; and positron emission tomographic imaging of Aβ, tau, and brain metabolism.^2^ Use of these biomarkers is hampered by a high degree of invasiveness, high costs, or limited availability.^5^ Blood-based biomarkers for AD may allow for efficient monitoring of disease processes in AD and could be used as a screening tool in primary care. One potential blood-based biomarker for AD is the neuronal injury marker neurofilament light (NFL)^6^ because patients with AD have increased CSF concentrations of NFL.^7^ Results from some studies^8^ suggest that patients with AD have increased plasma NFL concentrations. However, those studies were performed using standard immunoassay techniques with suboptimal analytical sensitivity to accurately quantify low abundant brain-specific proteins in blood samples.^10^ For this reason, our group has recently transferred the CSF NFL assay to an ultrasensitive single-molecule array (Simoa; Quanterix Corporation) platform, which provides an analytical sensitivity of 0.6 pg/mL compared with 78.0 pg/mL for the corresponding enzyme-linked immunosorbent assay (ELISA).^10^ Plasma NFL concentrations can be measured in all samples using the ultrasensitive single-molecule array and correlate closely with the corresponding CSF concentrations.^11^ Herein, we test this novel plasma NFL assay in patients with AD for the first time, to our knowledge. We studied cognitively healthy control individuals, patients with mild cognitive impairment (MCI) (MCI group), and patients with AD dementia (AD group) in a large prospective study. We tested the hypotheses that the plasma NFL concentration is increased in AD and that it correlates with impaired cognition, neuroimaging abnormalities, and CSF biomarkers of AD pathologic features.

## Methods

### ADNI Study Design

Data were obtained from the Alzheimer’s Disease Neuroimaging Initiative (ADNI) database (http://adni.loni.usc.edu). The ADNI was launched in 2003 as a public-private partnership, led by principal investigator Michael W. Weiner, MD (the most recent information on the ADNI is available at http://www.adni-info.org). The ADNI participants have been recruited from more than 50 sites across the United States and Canada. For the present study, we used data accessed from the Laboratory of Neuro Imaging (University of Southern California) ADNI database on October 6, 2016. The study data and samples were collected from September 7, 2005, to February 13, 2012. Regional ethical committees of all participating institutions approved the ADNI. All study participants provided written informed consent.

### ADNI Participants

Our ADNI cohort consisted of all cognitively healthy controls, patients with MCI, and patients with AD dementia with available baseline plasma NFL samples from the ADNI-1. Inclusion and exclusion criteria were described in detail previously.^13^ Briefly, all ADNI-1 participants were aged 55 to 90 years, had completed at least 6 years of education, were fluent in Spanish or English, and had no substantial neurological disease other than AD. Controls had Mini-Mental State Examination (MMSE) scores of 24 or higher, where lower scores indicate more impairment and higher scores less impairment (range, 0-30), and a Clinical Dementia Rating (CDR) score of 0, where lower scores indicate less impairment and higher scores more impairment (range, 0-3). Patients with MCI had MMSE scores of 24 or higher, objective memory loss tested by delayed recall of the Wechsler Memory Scale (WMS) logical memory II (>1 SD below the normal mean), a CDR score of 0.5, preserved activities of daily living, and absence of dementia. Patients with AD dementia fulfilled the National Institute of Neurological Communicative Disorders and Stroke–Alzheimer Disease and Related Disorders Association criteria for probable AD,^14^ had MMSE scores of 20 to 26, and had CDR scores of 0.5 to 1.0.

### Plasma NFL

Plasma NFL concentrations were measured using an NFL kit (NF-light; UmanDiagnostics), transferred onto the ultrasensitive single-molecule array platform using a home brew kit (Simoa Homebrew Assay Development Kit; Quanterix Corporation), as previously described.^15^ In the 14 analytical runs needed to complete the study, the relative error of the back-calculated concentrations was below 20% for all calibrators, run in triplicate, resulting in lower limits of quantifications of 2.2 ng/L and upper limits of quantification of 1620 ng/L. All samples measured within the range spanned by the limits of quantifications, and for the low-concentration quality control sample (14 ng/L), the intra-assay coefficient of variation was 11.0% and the interassay coefficient of variation was 11.1%. For the high-concentration quality control sample (137 ng/L), the corresponding coefficients of variation were 8.8% and 9.6%, respectively. The measurements were performed in September 2016 by a board-certified laboratory technician using a single batch of reagents.

### CSF Measurements

Cerebrospinal fluid was sampled by lumbar puncture from a subset of the participants, with CSF Aβ42, CSF total tau (t-tau), and CSF phosphorylated tau (p-tau) measured using a multiplex platform (xMAP; Luminex Corporation) with a kit (INNO-BIA AlzBio3; Fujirebio Europe). Participants were classified as Aβ positive or Aβ negative using a previously established cutoff (CSF Aβ42 < 192 ng/L).^16^ We excluded 8 patients with AD dementia who were Aβ negative and therefore likely to be misdiagnosed. The CSF NFL concentrations were measured using a commercial ELISA (NF-light; UmanDiagnostics) and have been reported previoushy.^7^ In total, we included CSF data from 112 controls, 189 patients with MCI, and 90 patients with AD dementia.

### Cognition

Cognition was assessed by MMSE, Alzheimer Disease Assessment Scale–cognitive subscale (ADAS-Cog 11), delayed recall of the WMS logical memory II, Trail-Making test part B (TMT-B), and Wechsler Adult Intelligence Scale–Revised (WAIS-R) digit symbol substitution test. All tests were administered at baseline and at 6, 12, 18, 24, 36, and 48 months, except for delayed recall of the WMS logical memory II, which was not assessed at 18 months.

### Neuroimaging

Structural brain images were acquired using 1.5-T MRI imaging systems with T1-weighted MRI scans using a sagittal volumetric magnetization-prepared rapid acquisition gradient echo sequence (at baseline and at 6, 12, 18, 24, 36, and 48 months). A software program (FreeSurfer; https://surfer.nmr.mgh.harvard.edu/) was used for quantification of cortical thickness and subcortical volumes.^17^ We used volumetric data for hippocampal volume and lateral ventricles (averaged between right and left sides). We used the mean cortical thickness for a set of regions defined a priori based on work by Jack et al^18^ to represent AD cortex (including entorhinal, inferior temporal, middle temporal, and fusiform cortex).

White matter hyperintensities (WMHs) were quantified at baseline and at 6, 12, 18, 24, 36, and 48 months using a fully automated protocol.^19^ Positron emission tomography with 18F-fluorodeoxyglucose image data were acquired at baseline and at 6, 12, 18, 24, 36, and 48 months.^20^ We created mean counts of the lateral and medial frontal, anterior, and posterior cingulate regions, as well as lateral parietal and lateral temporal regions.

### Statistical Analysis

We tested associations between plasma NFL and demographic factors using the Kruskal-Wallis test and Spearman rank correlation. We tested associations between biochemical markers and between plasma NFL and diagnosis using linear regression models. We calculated diagnostic accuracies using area under the receiver operating characteristic curve (AUROC) analysis with 10-fold cross-validated logistic regression models. We tested associations between plasma NFL concentrations and longitudinal cognition, brain structure, and brain metabolism using linear mixed-effects models. These models had random intercepts and slopes for time and an unstructured covariance matrix for the random effects and included the interaction between (continuous) time and plasma NFL as predictor. All outcome variables in linear mixed-effects models were standardized to *z* scores to facilitate comparisons between modalities. Therefore, β coefficients refer to standardized effects (β = 1 implies that an increase of 1 ng/L in plasma NFL was associated with a 1-SD increase in the dependent variable).

All tests were 2-sided. Statistical significance was set at *P* < .05. All regression analyses were corrected for age, sex, educational level, diagnosis, and *APOE* ε4 genotype, as well as intracranial volume for hippocampus and ventricles. All statistical analyses were performed using a software program (R, version 3.2.3; The R Foundation).

## Results

Table 1 lists demographics for the study population. In the whole cohort, plasma NFL correlated with age (Spearman ρ = 0.35, *P* < .001) but not with sex (median, 36.2 ng/L for men vs 37.4 ng/L for women; *P* = .98), educational level (ρ = −0.03, *P* = .52), or *APOE* ɛ4 genotype (37.7 ng/L in carriers vs 35.6 ng/L in noncarriers, *P* = .19). These results were similar within diagnostic groups, except that plasma NFL concentrations were higher in *APOE* ɛ4 carriers in the MCI group (35.9 ng/L in carriers vs 39.3 ng/L in noncarriers, *P* = .049) and in the AD dementia group (41.6 ng/L in carriers vs 51.5 ng/L in noncarriers, *P* = .03).

**Table 1.** Demographics for the Study Population^a^

Variable	Controls (n = 193)	MCI (n = 197)	AD Dementia (n = 180)	*P* Value
Age, mean (SD), y	75.9 (4.9)	74.7 (7.5)	75.3 (7.3)	.58
Female, No. (%)	87 (45.1)	65 (33.0)	86 (47.8)	.007
Educational level, mean (SD), y	16.0 (2.9)	15.8 (3.0)	14.7 (3.1)	<.001
*APOE* ɛ4 genotype carriers, No. (%)	50 (25.9)	103 (52.3)	123 (68.3)	<.001
Plasma NFL, mean (SD), ng/L	34.7 (21.4)	42.8 (29.0)	51.0 (26.9)	<.001
MMSE score, mean (SD)	29.1 (1.0)	26.9 (1.8)	23.2 (2.1)	<.001
CSF Aβ42, mean (SD), ng/L	207 (52)	165 (52)	134 (23)	<.001
Aβ+, No./total No. (%)	41/112 (36.6)	138/189 (73.0)	90/90 (100)	<.001
CSF t-tau, mean (SD), ng/L	68 (29)	102 (60)	126 (56)	<.001
CSF p-tau, mean (SD), ng/L	25 (15)	36 (19)	44 (20)	<.001

Abbreviations: Aβ, β-amyloid; AD, Alzheimer disease dementia; CSF, cerebrospinal fluid; MCI, mild cognitive impairment; MMSE, Mini-Mental State Examination; NFL, neurofilament light; p-tau, phosphorylated tau; t-tau, total tau.

^a^ *P* values are from the Kruskal-Wallis test or Fisher exact test. β-Amyloid positivity was defined as CSF Aβ42 less than 192 ng/L.

### Plasma NFL and Other Biochemical Markers in CSF and Plasma

**Figure 1.**
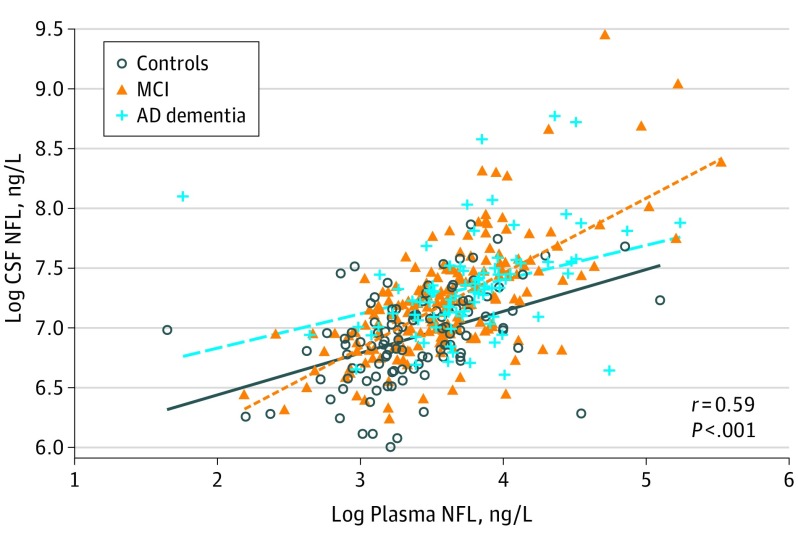
Plasma Neurofilament Light (NFL) and Cerebrospinal Fluid (CSF) NFL Fit lines are shown for individual diagnostic groups. The Spearman ρ and *P* values are for Spearman rank correlation in the whole cohort. Table 2 lists correlation data adjusted for covariates. AD indicates Alzheimer disease; MCI, mild cognitive impairment.

Plasma NFL correlated with high CSF NFL (Spearman ρ = 0.59, *P* < .001) (Figure 1) and with low CSF Aβ42, high CSF t-tau, high CSF p-tau, and high plasma tau (Table 2). The strongest correlations were seen with CSF NFL, which were also present in all diagnostic groups. Plasma NFL also correlated with CSF Aβ42 and CSF t-tau in the MCI group, as well as with plasma tau concentrations in all diagnostic groups.

**Table 2.** Correlations Between Plasma NFL and Other Biochemical Markers^a^

Biomarker	All Participants		Controls		MCI		AD Dementia	
	β Coefficient	*P* Value	β Coefficient	*P* Value	β Coefficient	*P* Value	β Coefficient	*P* Value
CSF NFL	0.480	<.001	0.371	<.001	0.615	<.001	0.242	.046
CSF Aβ42	−0.144	.01	0.002	.99	−0.243	<.001	−0.017	.87
CSF t-tau	0.125	.01	0.123	.21	0.170	.01	0.033	.76
CSF p-tau	0.105	.03	0.068	.49	0.113	.10	0.112	.31
Plasma tau	0.178	<.001	0.238	<.001	0.138	.03	0.188	.008

Abbreviations: AD, Alzheimer disease; CSF, cerebrospinal fluid; MCI, mild cognitive impairment; NFL, neurofilament light; p-tau, phosphorylated tau; t-tau, total tau.

^a^ Data are β coefficients (with *P* values) from linear regression models for correlations between plasma NFL and other biomarkers (all standardized to *z* scores), adjusted for age, sex, educational level, *APOE* ε4 genotype, and diagnosis. Models were tested in the whole cohort and in individual diagnostic groups.

### Plasma NFL in Different Diagnostic Groups

**Figure 2.**
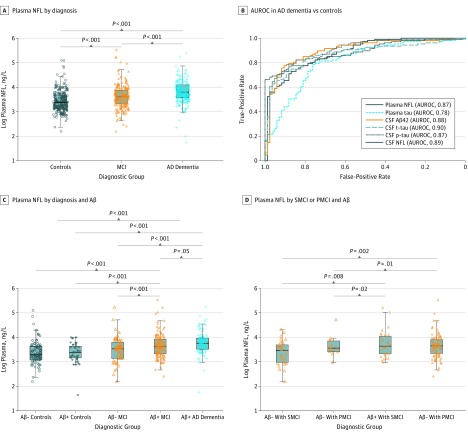
Plasma Neurofilament Light (NFL) by Diagnosis and Aβ A, Plasma NFL in controls, patients with mild cognitive impairment (MCI), and patients with Alzheimer disease (AD) dementia. B, Area under the receiver operating characteristic curve (AUROC) analyses for plasma NFL and other biomarkers to differentiate between the AD dementia group and controls. CSF indicates cerebrospinal fluid. C, Plasma NFL in controls, patients with MCI, and patients with AD dementia, stratified by occurrence of Aβ positivity (CSF Aβ42 < 192 ng/L). D, Plasma NFL in patients with stable MCI (SMCI) (no progression to dementia during ≥2 years’ follow-up) and patients with progressive MCI (PMCI) (conversion to dementia), with or without Aβ positivity. The models were adjusted for age, sex, educational level, and *APOE* ε4 genotype. Age retained an independent statistically significant association with higher plasma NFL (β = 0.025, *P* < .001 from the models shown in A). AB- indicates AB-negative; AB+, AB-positive.

Plasma NFL concentrations were higher in the AD group compared with controls and the MCI group, as well as in the MCI group compared with controls (Figure 2A). Plasma NFL differentiated between the AD dementia group and controls, with an AUROC of 0.87 (Figure 2B). By comparison, the AUROCs were 0.87 to 0.90 for CSF NFL, CSF Aβ42, CSF t-tau, and CSF p-tau and 0.78 for plasma tau. These AUROCs were corrected for age, sex, educational level, and *APOE* ε4 genotype. When only correcting for age, sex, and educational level, the AUROCs were reduced to 0.79 for plasma NFL, 0.81 for CSF NFL, 0.85 for CSF t-tau, 0.81 for CSF p-tau, and 0.64 for plasma tau.

### Plasma NFL and Aβ Pathologic Features

We compared plasma NFL between Aβ-negative controls, Aβ-positive controls, Aβ-negative patients with MCI, Aβ-positive patients with MCI, and (Aβ-positive) patients with AD dementia (Figure 2C). The AD dementia group had higher plasma NFL than Aβ-negative controls (mean, 48.8 vs 33.9 ng/L; *P* < .001), Aβ-positive controls (mean, 30.9 ng/L; *P* < .001), Aβ-negative MCI (mean, 38.1 ng/L; *P* < .001), and Aβ-positive MCI (mean, 44.5 ng/L; *P* = .05). There were no statistically significant differences between Aβ-negative and Aβ-positive controls and Aβ-negative patients with MCI.

### Plasma NFL and Progressive vs Stable MCI

Among the MCI group, 109 converted to AD dementia during follow-up, and 65 remained stable after at least 2 years’ follow-up. Twenty-three patients in the MCI group did not convert to AD dementia during follow-up but were observed for less than 2 years and were not included in the stable group. There was no difference in plasma NFL between Aβ-positive patients with progressive MCI and Aβ-positive patients with stable MCI, but both of these groups had higher plasma NFL than Aβ-negative patients with progressive MCI and Aβ-negative patients with stable MCI (Figure 2D).

### Plasma NFL and Cognition and Neuroimaging

**Figure 3.**
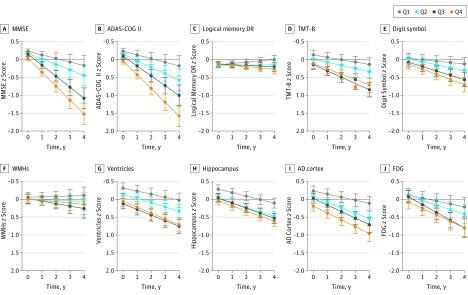
Associations Between Plasma Neurofilament Light and Cognitive and Neuroimaging Measures Data from linear mixed-effects models adjusted for age, sex, educational level, *APOE* ε4 genotype, and diagnosis, as well as intracranial volume for the neurofilament light quartiles for hippocampal volume and ventricular volume. ADAS-COG 11 indicates Alzheimer Disease Assessment Scale–cognitive subscale; AD cortex, Alzheimer disease cortex; Digit symbol, Wechsler Adult Intelligence Scale–Revised digit symbol substitution test; FDG, 18F-fluorodeoxyglucose; Logical memory DR, delayed recall of the Wechsler Memory Scale logical memory II; MMSE, Mini-Mental State Examination; Q, quartile; TMT-B, Trail-Making test part B; and WMHs, white matter hyperintensities.

Associations between plasma NFL and longitudinal cognitive and imaging measures are shown in Figure 3 (coefficients and *P* values are listed in the eTable in the Supplement). At baseline, high plasma NFL levels were associated with worse MMSE, ADAS-COG 11, and TMT-B scores and with larger ventricular volume, smaller hippocampal volume, and thinner cortices in the AD cortex region. Over time, high plasma NFL levels were associated with an accelerated decline in all measures, except for WMHs. The strongest influences were seen in MMSE (β = −0.073, *P* < .001 baseline and β = −0.116, *P* < .001 longitudinally) and ADAS-COG11 scores (β = 0.101, *P* < .001 baseline and β = 0.106, *P* < .001 longitudinally) for the cognitive measures and in AD cortex (β = −0.162, *P* < .001 baseline and β = −0.049, *P* < .001 longitudinally) for the imaging measures (details are provided in the eTable in the Supplement).

We also tested whether the influence of plasma NFL differed between diagnostic groups. Statistically significant interactions were found at baseline for the MCI group and MMSE score (β = −0.107, *P* = .008), ADAS-COG 11 score (β = 0.150, *P* = .003), delayed recall of the WMS logical memory II (β = −0.167, *P* < .001), TMT-B score (β = 0.283, *P* < .001), WAIS-R digit symbol substitution test score (β = −0.253, *P* = .002), and AD cortex (β = −0.282, *P* < .001), as well as for the AD group and MMSE score (β = −0.133, *P* = .002), ADAS-COG 11 score (β = 0.171, *P* = .001), delayed recall of the WMS logical memory II (β = −0.117, *P* = .01), and AD cortex (β = −0.178, *P* = .04). These results indicate that plasma NFL was more strongly correlated with the outcomes in the MCI group and the AD dementia group than in controls at baseline. Longitudinally, the only statistically significant interaction was for the MCI group and MMSE score (β = −0.107, *P* = .003), demonstrating that plasma NFL levels were more strongly correlated with longitudinal MMSE scores in the MCI group than in controls.

## Discussion

We present the first large study, to our knowledge, on plasma NFL in AD dementia. The main findings were that plasma NFL (1) correlated with CSF NFL independent of diagnosis, (2) was increased in the AD dementia group and in Aβ-positive patients with MCI, (3) had diagnostic accuracy for AD dementia in the same range as established CSF biomarkers, and (4) was associated with cognitive deficits and neuroimaging hallmarks of AD at baseline and during follow-up. Together, these findings support that plasma NFL is a promising biomarker for neuronal injury in AD, which may have potential for prognosis and monitoring of disease progression. This biomarker may be useful in clinical studies, in drug development, and ultimately in clinical practice. However, increased plasma NFL concentrations are also found in several other neurodegenerative disorders, such as progressive supranuclear palsy, frontotemporal dementia, and human immunodeficiency virus with brain engagement,^11^ meaning that it lacks disease specificity for AD. Therefore, we do not envision plasma NFL as a tool to differentiate AD from other neurodegenerative diseases. Rather, it may be valuable as a general biomarker for neurodegeneration.

Plasma NFL correlated with CSF NFL levels in the whole cohort and in diagnostic groups. Although these correlations were statistically significant, particularly in the whole cohort, the correlation coefficients were slightly lower than what was seen in previous studies of plasma or serum NFL levels, which included individuals with human immunodeficiency virus,^11^ progressive supranuclear palsy,^12^ other neurological diseases,^10^ and minor neurosurgical trauma.^21^ Hypothetically, it is possible that a greater variability in plasma NFL concentrations in AD compared with previously tested diseases could have influenced the correlations between plasma NFL and CSF NFL. Plasma NFL also correlated with other CSF biomarkers in the whole cohort, but those correlations were often not statistically significant within diagnostic groups, suggesting that they were confounded by diagnosis. This finding probably reflects that several different pathologic conditions are present in AD (eg, Aβ pathologic features, tau pathologic findings, and degeneration of different types of axons) and drive different biomarker responses, which will be weakly correlated overall. In MCI, a heterogeneous condition, high plasma NFL levles correlated with low CSF Aβ42 and high CSF t-tau, and this correlation supports the use of plasma NFL as a biomarker sensitive to AD-related biological changes in prodromal AD. Plasma NFL also correlated with plasma tau in all diagnostic groups. Although plasma tau appears to be a weaker biomarker for neuronal injury than plasma NFL,^22^ these measures may partly reflect the same process (eg, axonal degeneration).

The AD dementia group had higher plasma NFL levels than the MCI group, and the MCI group and the AD dementia group had higher plasma NFL levels than the controls. Although plasma NFL overlaps between the diagnostic groups, the accuracy of plasma NFL for AD dementia vs controls is close to established CSF AD biomarkers and much higher than for plasma tau (AUROC, 0.87 vs 0.78 when adjusted for demographics and *APOE* ε4; AUROC, 0.79 vs 0.64 when adjusted only for demographics). To our knowledge, this study represents the first time that a peripheral, noninvasive biomarker for neuronal injury has shown diagnostic accuracy for AD dementia comparable with established biomarkers in a large-scale multicenter cohort.

In the MCI group, plasma NFL levels were increased primarily in Aβ-positive patients with MCI (ie, prodromal AD). Plasma NFL concentration did not differ between clinically stable and progressive Aβ-positive patients with MCI. This result may have been influenced by the short follow-up time used to define stable MCI (2 years), which may be too abbreviated to verify the benign nature of the so-called stable condition.^23^ The finding that plasma NFL concentrations were increased already in prodromal AD is promising because it may render plasma NFL more useful in drug development, which is largely focused on this early stage of AD,^1^ as well as in clinical practice because patients with MCI increasingly seek medical evaluation. Plasma NFL did not differ between Aβ-positive and Aβ-negative controls, suggesting that any neuronal injury that may have occurred in Aβ-positive controls (ie, preclinical AD) is below the detection limit for plasma NFL. This result is well in line with the theory that preclinical AD is devoid of substantial neuronal injury.^24^

The final major finding was that plasma NFL was associated with several cognitive and imaging AD hallmarks at baseline and when those measures were analyzed over time. Specifically, plasma NFL was associated with general cognition (MMSE score and ADAS-COG 11 score) and executive function (TMT-B score) at baseline and with decline in all tested cognitive measures over time. The somewhat stronger correlations with timed tests, including TMT-B and WAIS-R digit symbol substitution test, compared with the memory test may suggest that plasma NFL primarily reflects damage to larger myelinated axonal processes of neurons. For imaging measures, associations were seen with lateral ventricles, hippocampal volume, and AD cortex thickness at baseline and over time, as well as with hypometabolism over time. The correlations were strongest in the MCI group and the AD dementia group but were statistically significant in the whole cohort when adjusting for diagnosis.

### Limitations

This study is limited by the lack of patients with neurodegenerative diseases other than AD, which prevented our testing for disease specificity of plasma NFL. Another limitation is the restricted sample in the ADNI, such that patients with substantial vascular burden were excluded. This exclusion may have made it difficult to detect subtle associations between plasma NFL and white matter pathologic findings and may explain the surprising finding that plasma NFL did not correlate with WMHs, despite that CSF NFL has been considered a marker of white matter pathologic features,^26^ including in AD.^7^ Future studies should test plasma NFL in a more unselected group of patients with AD and may also explore different proxies for white matter injury. A larger age span should also be included in future work because it is possible that vascular comorbidities may change with age and alter the diagnostic accuracy of plasma NFL for patients with AD vs controls and other diseases.

All main results were in the expected directions, without illogical data. The main findings that plasma NFL concentration was increased in the AD dementia group and correlated with CSF NFL, imaging, and cognitive hallmarks of AD were all statistically significant. We believe that the consistency of these data makes it unlikely that they were falsely positive. Therefore, we reported *P* values uncorrected for multiple comparisons.

## Conclusions

We found that plasma NFL concentration is increased in AD, even in prodromal disease, and that it correlates with important disease hallmarks, measured by cognitive tests, neuroimaging, and CSF biomarkers. The fact that plasma NFL concentration is also elevated in other neurological diseases^11^ and that NFL may be released from neurons in Aβ-dependent and Aβ-independent pathologic conditions^27^ argues against the use of plasma NFL for differential diagnosis of AD vs other dementias. However, plasma NFL may be a valuable noninvasive tool to assess neurodegeneration and to identify individuals at risk for future cognitive decline and brain atrophy. Therefore, plasma NFL is a promising peripheral biomarker for neurodegeneration, including in AD. In a clinical trial scenario, it is possible that plasma NFL may be used (together with demographics and *APOE* ε4 genotype data) to predict longitudinal disease progression. Future studies with repeated samples should test plasma NFL as a longitudinal noninvasive proxy for neurodegeneration.

## Notes

Supplement. eTable. Associations Between Plasma NFL and Cognition and Neuroimaging
