# Supplementary material for: Association of Plasma Neurofilament Light With Neurodegeneration in Patients With Alzheimer Disease
Source: JAMA Neurol. 2017 Mar 27;74(5):557–66. doi: 10.1001/jamaneurol.2016.6117 (PMC5822204; doi:10.1001/jamaneurol.2016.6117)
Supplement: Supplement. — eTable. Associations Between Plasma NFL and Cognition and Neuroimaging [file jamaneurol-74-557-s001.pdf]

Supplementary Online Content

Mattsson N, Andreasson U, Zetterberg H, Blennow K; Alzheimer's Disease Neuroimaging Initiative. Association of plasma neurofilament light with neurodegeneration in patients with Alzheimer disease. *JAMA Neurol*. Published online March 27, 2017. doi:10.1001/jamaneurol.2016.6117

eTable. Associations Between Plasma NFL and Cognition and Neuroimaging

This supplementary material has been provided by the authors to give readers additional information about their work.

.

© 2017 Mattsson N et al. *JAMA Neurology*.

eTable. Associations Between Plasma NFL and Cognition and Neuroimaging

	**Baseline**		**Longitudinally**	
**Outcome**	**β**	**p-value**	**β**	**p-value**
**MMSE**	**-0.0725**	**<0.001**	**-0.116**	**<0.001**
**ADAS-cog**	**0.101**	**<0.001**	**0.106**	**<0.001**
**Logical Memory delayed recall**	0.00032	0.99	**-0.0269**	**<0.001**
**TMT-B**	**0.0823**	**0.023**	**0.0380**	**0.0012**
**Digit Symbol**	-0.0692	0.056	**-0.0378**	**<0.001**
**White matter hyperintensities**	0.00437	0.90	0.0291	0.12
**Lateral ventricles**	**0.145**	**<0.001**	**0.0319**	**<0.001**
**Hippocampal volume**	**-0.124**	**<0.001**	**-0.0189**	**<0.001**
**AD-cortex thickness**	**-0.162**	**<0.001**	**-0.0489**	**<0.001**
**FDG**	-0.0846	0.10	**-0.0474**	**<0.001**

Results from linear mixed effects models, adjusted for age, sex, education, *APOE* ε4, diagnosis, and (for ventricle volume and hippocampal volume) intracranial volume. All outcome measures were standardized to z-scores to facilitate comparisons between models.

© 2017 Mattsson N et al. *JAMA Neurology*.
